# The global burden and attributable risk factor analysis of cardiovascular disease between females and males, 1990–2021: findings from the 2021 global burden of disease study

**DOI:** 10.1097/JS9.0000000000003397

**Published:** 2025-09-23

**Authors:** Jiaqi Fan, Jun Chen, Litao Wang, Changzheng Yuan, Xianbao Liu, Jian’An Wang

**Affiliations:** a Department of Cardiology, Second Affiliated Hospital Zhejiang University School of Medicine, Hangzhou, People’s Republic of China; b State Key Laboratory of Transvascular Implantation Devices, Hangzhou, People’s Republic of China; c Cardiovascular Key Laboratory of Zhejiang Province, Hangzhou, People’s Republic of China; d Department of Vascular Surgery, Second Affiliated Hospital Zhejiang University School of Medicine, Hangzhou, People’s Republic of China; e School of Public Health, Zhejiang University School of Medicine, Hangzhou, People’s Republic of China; f Department of Nutrition, Harvard T. H. Chan School of Public Health, Boston, MA, USA

**Keywords:** attributable risk factor, cardiovascular disease, disability-adjusted life years, global burden of disease, sex difference

## Abstract

**Background:**

Cardiovascular disease (CVD) is the leading cause of morbidity and mortality globally yet remains under-recognized and under-researched in women. Despite increasing emphasis on sex and gender influences in CVD, there is limited and conflicting evidence on sex differences in the burden and risk factors of CVD worldwide. We aimed to assess sex differences in the global burden of CVD and its attributable risk factors from 1990 to 2021.

**Material and methods:**

Global Burden of Disease (GBD) 2021 examined sex differences in CVD incidence, prevalence, mortality, and disability-adjusted life years (DALYs), including ischemic heart disease, stroke, cardiomyopathy, and others. We evaluated absolute, rate-based, and age-standardized measures, as well as sex-specific risk factor burdens.

**Results:**

Age-standardized incidence, prevalence, mortality, and DALY rates of CVD were consistently higher in males compared with females across most CVD types. Ischemic heart disease showed the largest disparities, with males having higher incidence (147.9 [95% uncertainty interval 57.7–244.6] per 100 000), prevalence (1253.6 [633.1–1816.6] per 100 000), mortality (51.0 [38.3–63.1] per 100 000), and DALY rates (1286.2 [1069.9–1511.7] per 100 000) compared to females. In contrast, females had higher incidence (55.9 [33.9–79.6] per 100 000) and prevalence rates (699.5 [415.5–983.5] per 100 000) compared to males in lower extremity peripheral arterial disease. A stable difference between females and males was observed in age-standardized DALY rates for overall CVD between 1990 and 2021. High systolic blood pressure, dietary risks, and air pollution were major contributors to CVD-related DALYs for both sexes, although tobacco use (144.0 million DALYs for males vs. 31.5 million for females), elevated LDL cholesterol (130.1 million DALYs for males vs. 75.9 million for females), and impaired kidney function (59.8 million DALYs for males vs. 39.3 million for females) were disproportionately higher in males.

**Conclusion:**

The global burden of CVD is substantially higher in males compared with females, particularly for ischemic heart disease. While primary risk factors are shared, males show a greater burden from tobacco use and elevated LDL cholesterol.

**HIGHLIGHTS**

- Males have consistently higher rates of CVD than females, especially for ischemic heart disease.
- Women have a higher incidence and prevalence of lower extremity peripheral arterial disease compared to men.
- Major CVD risk factors such as high blood pressure, diet, and air pollution affect both sexes similarly.
- Tobacco use and elevated LDL cholesterol are disproportionately higher contributors to CVD in men.
- The global burden of CVD in males is significantly higher, particularly for ischemic heart disease, compared to females.

## Introduction

Cardiovascular disease (CVD) is the leading cause of morbidity and mortality worldwide[1]. Historically perceived as a predominantly male disease, CVD remains understudied, under-recognized, underdiagnosed, and undertreated in women[2]. Despite an emphasis on the role of sex (biological constructs) and gender (social constructs) in the management of CVD, the differences in global burden and its attributable risk factors between females and males have received little attention and controversial evidence.

Although women are generally considered to have a lower prevalence of CVDs than men, previous data analysis from the Global Burden of Disease (GBD) study showed that women had a higher prevalence rate of CVDs than men among youths and young adults aged 15–39 years. Conversely, men had higher rates of disability-adjusted life years (DALYs) and mortality than women in this age group[3]. In 2019, total CVD DALYs were greater in men than in women up to the age of 80–84 years, after which the pattern reversed. The sex differences in DALYs were most pronounced between the ages of 30 and 60 years (with men being higher) and over 80 years (with women being higher[4].

Although previous GBDs studies have presented global burden of CVD and risk factors from 1990 to 2019, there has yet to be a systematic, comparable quantification of sex differences for these different CVDs, along with the contribution of attributable risk factors[4]. Existing studies, primarily from high-income countries, have reported that hypertension, diabetes, and smoking are more strongly associated with ischemic heart disease in women than in men.^[5–8]^ However, the other global study, mainly from low-income and middle-income countries, did not observe significant sex difference for the association of diabetes and smoking with major cardiovascular disease, a composite of cardiovascular disease death, myocardial infarction (MI), stroke, and heart failure[9].

We utilized data from the GBD 2021 study to investigate differences between females and males in the incidence, prevalence, mortality, and DALYs of CVDs globally. Our analysis spans from 1990 to 2021 and includes assessments in terms of number, rate, and age-standardized rate. Additionally, we estimated the differences in attributable risk factors for CVDs from 1990 to 2021.

The work has been reported in line with the Strengthening Reporting Of Cohort Studies in Surgery (STROCSS) criteria[10].

## Materials and methods

### The GBD 2021 and study participants

The GBD 2021 study provides a comprehensive overview of the causes of death, disability, and associated risk factors on a global scale. It delivers detailed estimates of mortality, incidence, prevalence, years of life lost (YLLs), years lived with disability (YLDs), and disability-adjusted life years (DALYs) from 1990 to 2021, covering 204 countries and territories and 371 diseases and injuries[1]. In this study, we leverage these estimates to explore gender differences in cardiovascular disease, focusing on prevalence, incidence, DALYs, and attributable risk factors between females and males. The GBD adheres to the Guidelines for Accurate and Transparent Health Estimates Reporting and is approved by the University of Washington Institutional Review Board. No individual-level data was used, and all estimates are aggregated at national and regional levels, ensuring the protection of personal health information.

In this study, we analyzed the impact of sex and gender on CVDs using the GBD 2021 data. We extracted information on the prevalence, incidence, and DALYs associated with overall and type-specific CVDs, including rheumatic heart disease, ischemic heart disease, stroke, hypertensive heart disease, non-rheumatic valvular heart disease, cardiomyopathy and myocarditis, atrial fibrillation and flutter, aortic aneurysm, and endocarditis. Additionally, we assessed associated risk factors.

GBD data provide valuable insights but mainly differentiate by sex, often conflating sex and gender, limiting our ability to separate biological and gender identity effects on cardiovascular health. Despite this, our analysis examines differences in CVD burden between females and males, acknowledging influences from both biological and gender-related factors.

### Estimation framework for the disease burden of CVDs

In this study, overall and type-specific CVDs, along with related mortality, were identified based on standardized definitions reported in prior literature. For GBD 2021, the Bayesian meta-regression tool DisMod-MR 2.1 estimated incidence and prevalence of CVDs using data from surveys, cohort studies, registries, and health system records. Mortality data were obtained from vital registration sources using ICD codes, and CVD-related mortality was estimated with the Cause of Death Ensemble Model. Disability-adjusted life years represent the total burden of disease by combining early death and time lived with disability. DALYs were calculated by combining years of life lost (YLLs) and years lived with disability (YLDs), where YLLs were derived from deaths and YLDs from disease prevalence and duration, weighted by disability severity. Temporal trends in CVD burden—including incidence, prevalence, mortality, and DALYs—were analyzed across the full timeframe from 1990 to 2021 to capture both historical and contemporary patterns.

### Estimated burden of sex-specific CVDs attributable to risk factors

The GBD 2021 study evaluates 87 risk factors across various dimensions of health, including specific and aggregate risk factors at global, regional, and national levels for 204 countries and territories. In the GBD study, risk is an attribute, behavior, exposure, or other factors causally associated with an increased probability of developing a disease or experiencing an injury. The risk hierarchy in the GBD comparative risk assessment (CRA) is structured into four-levels of risk factors. For this study, the attributable DALYs for CVDs were derived from the risk factors in levels 3 and 4, including factors like ambient air pollution, unhealthy diets, high body mass index, high blood pressure, smoking, and kidney dysfunction. Definitions of all risk factors are available on the Global Health Data Exchange website. To ensure the analysis reflects the most contemporary epidemiological landscape, the year 2021 was selected for the primary assessment of attributable risk factors.

### Statistical analysis

Using data from the GBD 2021 analytical tools, we conducted a comprehensive analysis of both the absolute and relative differences in prevalence, incidence, and DALY rates between females and males for overall and specific CVD types. Absolute differences were calculated by subtracting the male DALY rate from the corresponding female DALY rate, stratified by age, cause, super-region, and year. A positive absolute difference indicates a higher rate among females. For the relative difference, we calculated the percent difference by dividing the absolute difference by the male DALY rate and multiplying by 100. A positive relative difference suggests higher rates in females compared to males.

Each metric, including the absolute and relative differences and changes over time, was computed at the draw level using 500 draws from the posterior distribution. The results are reported with 95% uncertainty intervals (UIs), based on the 2.5th and 97.5th percentiles of the draws. These UIs offer a measure of uncertainty around the estimates, indicating the range within which the true values likely fall. We assessed the presence of a statistically significant difference between females and males by checking whether the 95% UIs for the differences excluded zero, which would suggest the existence of a meaningful difference.

## Results

### Overall CVD burden between female and male

The age-standardized incidence and prevalence rates of CVD were found to be higher in males compared to females (Table 1). Among the 14 specific types of CVDs, 5 exhibited both higher global age-standardized incidence and prevalence rates in males: ischemic heart disease, intracerebral hemorrhage, non-rheumatic calcific aortic valve disease, non-rheumatic degenerative mitral valve disease, and cardiomyopathy/myocarditis. Endocarditis showed a higher incidence rate in males, while ischemic stroke had a higher prevalence in males. The largest absolute difference in age-standardized incidence rates was observed for ischemic heart disease, with a significant difference of −147.9 (95% UI −244.6 to −57.7) cases per 100 000, favoring females. Similarly, the largest difference in prevalence rates was also for ischemic heart disease, with −1253.6 (95% UI −1816.6 to −633.1) cases per 100 000 fewer in females than males, highlighting the substantial burden of ischemic heart disease on the male population. Among the 14 causes examined, only one, lower extremity peripheral arterial disease, demonstrated both higher age-standardized incidence and prevalence rates in females compared to males. Specifically, females had an estimated 55.9 (95% UI 33.9–79.6) more cases per 100 000 for incidence and 699.5 (95% UI 415.5–983.5) more cases per 100 000 for prevalence compared to males. Additionally, subarachnoid hemorrhage and pulmonary arterial hypertension appeared to have higher prevalence rates among females than males. More details in age-standardized incidence and prevalence rates of CVD between females and males in different super-regions were presented in Supplementary Digital Content Tables S1–7, available at: http://links.lww.com/JS9/F137 (appendix pp 3–16).

**Table 1:** Age-standardized incidence and prevalence rates of selected cardiovascular disease subcategories for females and males and their absolute and relative percentage differences globally in 2021

	Age-standardized incidence rate, per 100 000 (95% UI)				Age-standardized prevalence rate, per 100 000 (95% UI)			
	Females	Males	Absolute differences	Relative percentage difference	Females	Males	Absolute difference	Relative percentage difference
All CVD	713 (654.6–777.9)	866.8 (788.5–950)	−154 (−261.9 to −51.8)	−17.6 (−28.5 to −6.5)	6750.6 (6298.7–7163.2)	7666.9 (7159.9–8166.6)	−910.7 (−1580.5 to −287.9)	−11.8 (−20 to −3.9)
Rheumatic heart disease	55.1 (43.6–68.7)	46.5 (36.5–57.8)	8.5 (−8.9 to 26)	20 (−16.5 to 67.9)	754.8 (600.8–936.7)	614.2 (482.6–761.3)	154.5 (−62.8 to 386.4)	27.6 (−8.7 to 76.5)
Ischemic heart disease	301.6 (248.6–360.7)	450.4 (373.7–534.6)	−147.9 (−244.6 to −57.7)	−32.3 (−48.5 to −14.7)	2357.6 (2063.3–2751.9)	3610.2 (3153.1–4165)	−1253.6 (−1816.6 to −633.1)	−34.4 (−46.1 to −20.1)
Ischemic stroke	82.8 (71.3–94.8)	102.8 (88.7–118.8)	−19.2 (−39.3 to 0.9)	−18.3 (−34.7 to 1)	769.4 (713–826)	881.9 (818.6–944.5)	−112 (−200.2 to −28.4)	−12.6 (−21.4 to −3.4)
Intracerebral hemorrhage	33.6 (29.5–37.2)	48.7 (43.1–54.3)	−15.1 (−22.4 to −8.5)	−30.8 (−41.6 to −18.9)	166.1 (152.2–181.2)	225.1 (204.9–245.6)	−58.7 (−83.4 to −35.7)	−25.9 (−35 to −17.1)
Subarachnoid hemorrhage	8.2 (7.2–9.4)	8.5 (7.5–9.6)	−0.3 (−1.8 to 1.2)	−2.9 (−19.6 to 15.6)	97.9 (89.7–106.6)	85.5 (77.7–93.7)	12.8 (1.2–24.2)	15.3 (1.3–30.6)
Hypertensive heart disease					146.6 (117–183.7)	148.9 (116.4–189.1)	−3.7 (−49.6 to 47.1)	−0.8 (−29.7 to 40.5)
Non-rheumatic calcific aortic valve disease	10.2 (8.7–11.5)	14 (12.2–15.9)	−3.9 (−6.3 to −1.6)	−27.2 (−41.1 to −13)	128.9 (110.1–147.6)	193.2 (166.6–220.4)	−64.7 (−101.3 to −29.9)	−33 (−46.8 to −17.8)
Non-rheumatic degenerative mitral valve disease	8.7 (8.1–9.2)	17.7 (16.5–19)	−9 (−10.5 to −7.6)	−50.9 (−55.6 to −46)	121.1 (112.9–129.8)	257.9 (241–278.2)	−136.4 (−155.2 to −116.3)	−52.9 (−57.5 to −47.8)
Cardiomyopathy and myocarditis	13.3 (10.7–16.2)	19.2 (15.6–23.5)	−5.9 (−10.5 to −1.1)	−29.9 (−48.5 to −6.7)	54.2 (45–64)	78.3 (65.4–90.9)	−23.8 (−39.6 to −7.8)	−29.8 (−44.9 to −11.9)
Pulmonary arterial hypertension	0.5 (0.4–0.7)	0.5 (0.4–0.6)	0.1 (−0.1 to 0.2)	15.1 (−14.6 to 52.4)	2.8 (2.2–3.4)	1.8 (1.4–2.2)	1 (0.3–1.6)	55.7 (14.5–102.3)
Atrial fibrillation and flutter	47.3 (37.4–60.9)	57.1 (46.2–72.1)	−9.2 (−28.9 to 7)	−15.1 (−42.8 to 14.8)	529.1 (430.8–663.1)	728.9 (601.9–895.8)	−194.4 (−386.6 to 2)	−25.8 (−46.2 to 0.4)
Lower extremity peripheral arterial disease	141.2 (122–162.2)	86.6 (75.4–99.6)	55.9 (33.9–79.6)	66.3 (35.2–104.1)	1643 (1425.9–1893.3)	953.5 (830.8–1098.2)	699.5 (415.5–983.5)	74.6 (40–114.1)
Endocarditis	10.6 (9.1–12.3)	14.8 (12.8–17.1)	−4.3 (−7.3 to −1.5)	−28.5 (−43.7 to −11.7)	5 (4.3–5.7)	5.7 (4.9–6.5)	−0.7 (−1.9 to 0.4)	−11.7 (−30.2 to 7.8)
Other cardiovascular and circulatory diseases					866.3 (680.5–1075.9)	1109.1 (859.2–1400.9)	−244.3 (−549.9 to 74.7)	−20.7 (−41.7 to 8.1)

The age-standardized mortality and DALY rates for all CVDs were higher in males compared to females (Table 2). Ischemic heart disease, given its overall global burden, accounted for the largest absolute difference between the sexes in 2021, with males experiencing 51 (95% UI 38.3–63.1) more deaths and 1286.2 (95% UI 1069.9–1511.7) more DALYs per 100 000 than females. Intracerebral hemorrhage had the second-largest disparity, with global mortality rates of 32.1 (95% UI 28.3–36) per 100 000 for females and 47.4 (95% UI 42.3–53.2) for males. Similarly, the global DALY rates for intracerebral hemorrhage were 742.5 (95% UI 657.6–822.7) for females and 1123.1 (95% UI 1014.1–1251.7) for males, further underscoring the disproportionate impact on males. Ischemic stroke ranked third in terms of absolute and relative percentage differences, followed by cardiomyopathy and myocarditis. No CVD conditions demonstrated higher mortality or DALY rates in females compared to males. More details in age-standardized mortality and DALY rates of CVD between females and males in different super-regions were presented in Supplementary Digital Content Table S8–14, available at: http://links.lww.com/JS9/F137 (appendix pp 17–30).

**Table 2:** Age-standardized mortality and DALY rates of selected cardiovascular disease subcategories for females and males and their absolute and relative percentage differences globally in 2021

	Age-standardized mortality rate, per 100 000 (95% UI)				Age-standardized DALY rate, per 100 000 (95% UI)			
	Females	Males	Absolute differences	Relative percentage difference	Females	Males	Absolute difference	Relative percentage difference
All CVD	196.7 (175.3–213.2)	281.1 (259.8–301.1)	−84.7 (−112.5 to −56.2)	−30 (−38.2 to −21)	4048.2 (3721.4–4327)	6169 (5774.3–6613.8)	−2119.8 (−2636.6 to −1609.8)	−34.3 (−40.7 to −27.6)
Rheumatic heart disease	4.7 (4–5.8)	4.2 (3.5–6.2)	0.5 (−1.3–2)	14.2 (−22.9 to 65.1)	174 (148.2–211.1)	150.1 (125.2–205)	22 (−27.1–72.4)	16.7 (−14.2 to 60.9)
Ischemic heart disease	85.3 (75.9–92.3)	136.8 (127.4–145.9)	−51 (−63.1 to −38.3)	−37.3 (−44.2 to −29.7)	1596.1 (1464–1706.9)	2890.7 (2714.8–3091.3)	−1286.2 (−1511.7 to −1069.9)	−44.5 (−50.1 to −38.9)
Ischemic stroke	38.5 (33.5–42.5)	51.2 (46.2–56.2)	−12.4 (−19.3 to −6)	−24.1 (−35.4 to −12.3)	719.5 (642.8–791.6)	975.3 (885.6–1069.8)	−259.4 (−378.5 to −146.5)	−26.4 (−35.6 to −16.3)
Intracerebral hemorrhage	32.1 (28.3–36)	47.4 (42.3–53.2)	−15.3 (−21.7 to −8.4)	−32.2 (−43.1 to −19.8)	742.5 (657.6–822.7)	1123.1 (1014.1–1251.7)	−378.6 (−520.4 to −238.2)	−33.6 (−43.6 to −23.6)
Subarachnoid hemorrhage	3.9 (3.4–4.5)	4.5 (3.6–5.6)	−0.6 (−1.6 to 0.4)	−11.4 (−30.9 to 10.7)	116.4 (104.2–133.1)	134.1 (109.9–167.9)	−17.2 (−53.2 to 16.9)	−11.7 (−32.8 to 15.3)
Hypertensive heart disease	16.5 (13.4–18.8)	15.9 (12.4–18.3)	0.8 (−3.4 to 4.7)	5.9 (−18.7 to 34.7)	299.3 (235.4–336.1)	301.4 (240.9–345.2)	−1.6 (−78 to 73.6)	0.3 (−23 to 28.7)
Non-rheumatic calcific aortic valve disease	1.7 (1.4–1.9)	1.9 (1.7–2)	−0.1 (−0.5 to 0.2)	−7.3 (−23.3 to 9.7)	24.4 (20.7–27.4)	31.1 (28.4–33.9)	−6.6 (−10.9 to −2.1)	−21.1 (−33.4 to −7.4)
Non-rheumatic degenerative mitral valve disease	0.5 (0.4–0.6)	0.4 (0.4–0.5)	0.1 (0–0.2)	19 (−4.1 to 45.1)	11.3 (9.2–13.6)	11.6 (9.7–14.1)	−0.3 (−3.3 to 3.1)	−1.2 (−25.9 to 30.4)
Cardiomyopathy and myocarditis	3.5 (3–3.8)	6.5 (5.9–7.1)	−3 (−3.7 to −2.4)	−46.3 (−53.6 to −39.3)	94.1 (85.1–102.6)	192.5 (173.7–211.4)	−98.8 (−117.8 to −78.1)	−51.1 (−57.2 to −44.6)
Pulmonary arterial hypertension	0.3 (0.2–0.3)	0.3 (0.2–0.3)	0 (−0.1 to 0.1)	4.2 (−21.4 to 36.5)	8.4 (6.9–10.5)	8.1 (6.7–9.4)	0.4 (−1.9 to 2.7)	6.3 (−22.3 to 37.8)
Atrial fibrillation and flutter	4.3 (3.5–4.8)	4.4 (3.9–4.8)	−0.1 (−0.8 to 0.6)	−2.5 (−18.2 to 14.9)	92.2 (76.8–111.2)	112 (93.3–135.3)	−20.3 (−48.6 to 7.4)	−17.2 (−37.4 to 7.8)
Lower extremity peripheral arterial disease	0.7 (0.6–0.8)	1 (0.9–1.1)	−0.3 (−0.4 to −0.1)	−25.7 (−38.9 to −12.1)	17.2 (13–24.3)	20 (17.3–24.2)	−2.9 (−9.3 to 3.4)	−13.9 (−44.1 to 18.4)
Endocarditis	0.8 (0.7–1)	1.1 (1–1.2)	−0.3 (−0.5 to −0.1)	−25 (−39 to −9)	20.6 (16.4–23.7)	30.6 (25.7–35.4)	−10 (−16 to −4.1)	−32.1 (−46.2 to −14.9)
Other cardiovascular and circulatory diseases	2.5 (2.2–2.8)	3 (2.7–3.3)	−0.5 (−0.9 to 0)	−16.4 (−28.8 to −1.8)	108.5 (91.2–130.2)	136.2 (114.7–166.6)	−27.7 (−57.9 to 1.9)	−19.6 (−37.7 to 1.5)

### Trends of CVD burden over time between female and male

**Figure 1.**
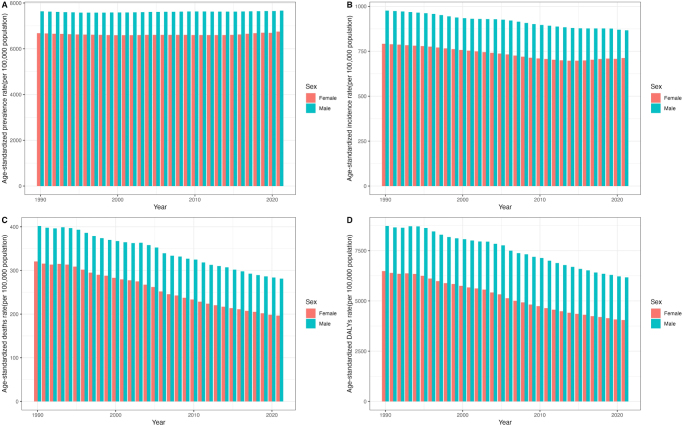
Temporal trends in age-standardized prevalence (A), incidence (B), mortality (C), and DALY (D) rates for overall CVD between female and male from 1990 to 2021.

**Figure 2.**
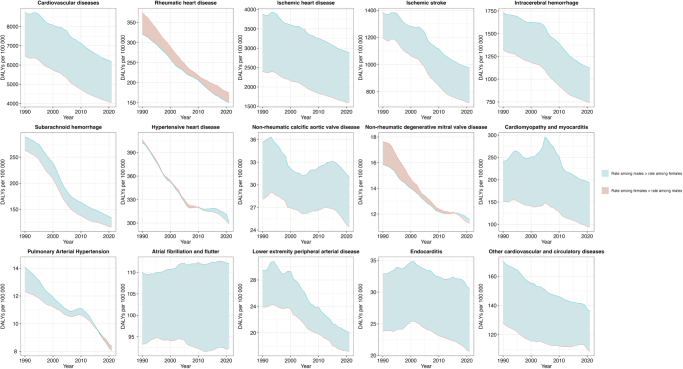
Trends of all CVD and subtypes burden over time between female and male (DALYs, age-standardized).

Temporal trends in age-standardized prevalence, incidence, mortality, and DALY rates for overall cardiovascular disease between females and males from 1990 to 2021 were presented in Figure 1. Though the rates decreased over time for both female and male, we observed a stable absolute difference between females and males in age-standardized DALY rates for overall cardiovascular disease between 1990 and 2021 (Fig. 2). Gradual changes in differences were evident in the two conditions where the absolute difference between males and females decreased, including for rheumatic heart disease and lower extremity peripheral arterial disease. The rheumatic heart disease showed significantly higher DALY rates in females compared to males (174.0 [95% UI 148.2–211.1] vs. 150.1 [125.2–205.0]), while the lower extremity peripheral arterial disease showed a higher DALY rate in males (17.2 [13.0–24.3] vs. 20.0 [17.3–24.2]). The age-standardized DALY rates for non-rheumatic degenerative mitral valve disease declined for both sexes. Initially, females had higher rates (17.6 [14.9–20.6] vs. 15.9 [13.3–18.9]), but the gap narrowed over time, and males eventually exhibited a higher burden (11.3 [9.2–13.6] vs. 11.6 [9.7–14.1]). In contrast, the age-standardized DALYs for pulmonary arterial hypertension were initially higher in males than females (14.1 [11.9–16.4] vs. 12.3 [7.8–16.8]), but eventually exhibited lower values in males than females over time (8.1 [6.7–9.4] vs. 8.4 [6.9–10.5]). More details in trends of CVD prevalence, incidence, and mortality rates over time between females and male were presented in Supplementary Digital Content Figures S1–3, available at: http://links.lww.com/JS9/F137 (appendix pp 31–33).

### Burden of CVD between female and male by age

**Figure 3.**
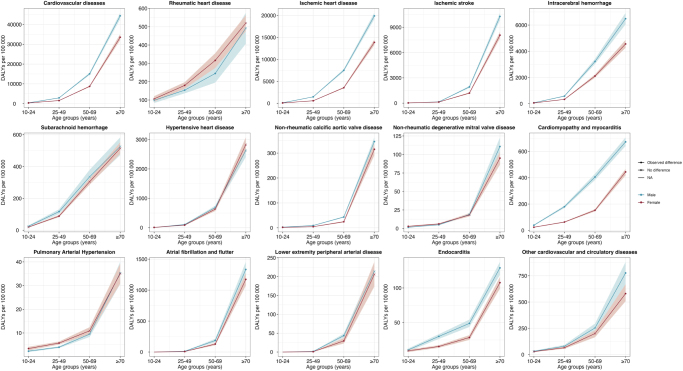
DALYs of cardiovascular disease between female and male by age.

Overall, DALYs for all cardiovascular conditions increased with advancing age, with the most significant increase observed in the ≥70 years group (Fig. 3). Ischemic heart disease showed the highest burden among cardiovascular conditions, particularly in individuals aged 50 years and older, with males consistently demonstrating higher DALYs than females across all age groups. Notably, the difference between males and females was most pronounced in ischemic heart disease, ischemic stroke, and intracerebral hemorrhage, where males had substantially higher DALYs, especially in older age groups. Atrial fibrillation or flutter, cardiomyopathy or myocarditis, and endocarditis also displayed age-related increases in DALYs, though the sex differences were generally less pronounced compared to ischemic heart disease. Rheumatic heart disease showed a higher burden in females compared to males in aged 25–69 years, while males showed a rapid increase in individuals aged ≥70 years. Conditions such as non-rheumatic calcific aortic valve disease and non-rheumatic degenerative mitral valve disease exhibited a substantial increase in DALYs among individuals aged ≥70 years, while the former showed higher burdens in males in all age categories and the latter presented a comparable burden between males and females in age less than 70 years. Interestingly, pulmonary arterial hypertension demonstrated slightly higher DALYs in females compared to males, particularly in the younger age groups. More details in burden of CVD prevalence, incidence, and mortality rates between females and males by age were presented in Supplementary Digital Content Figures S4–6, available at: http://links.lww.com/JS9/F137 (appendix pp 34–36).

### Burden of CVD between female and male in different region

**Figure 4.**
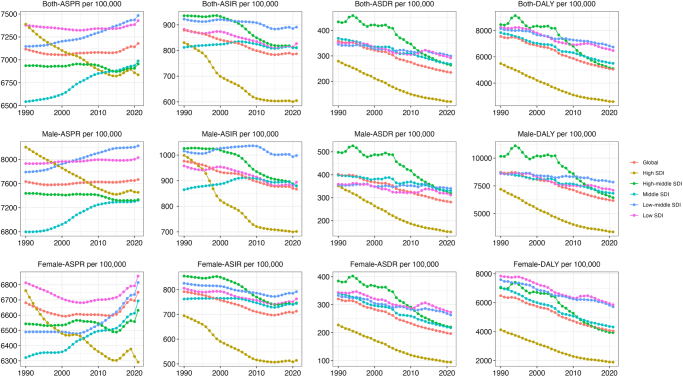
Trends in cardiovascular disease prevalence, incidence, deaths and disability-adjusted life-years from 1990 to 2021.

Trends in CVD metrics (ASPR, ASIR, ASDR, and DALY per 100 000) from 1990 to 2020 across different socio-demographic index (SDI) levels are shown in Figure 4. For both sexes, age-standardized prevalence rates (ASPR) per 100 000 have shown variable trends across SDI regions from 1990 to 2020, with high SDI countries showing an obvious reduction, while middle SDI and low-middle SDI regions have exhibited increasing trends. Age-standardized incidence rates (ASIR) per 100 000 reveal a somewhat similar pattern, with high SDI countries demonstrating a steady decline for both sexes. In contrast, middle and low-middle SDI regions showed fluctuating trends. Age-standardized mortality rates (ASDR) and DALYs per 100 000 have decreased across all SDI regions for both sexes, with high SDI regions experiencing the most significant reduction. In contrast, regions with lower SDI exhibit relatively higher ASDR and DALY, though they also show a downward trend over the period. The observed pattern of rate changes from 1990 to 2020 in these four metrics did not show significant differences between males and females.

### CVD-related DALYs attributable to risk factors between females and males

**Figure 5.**
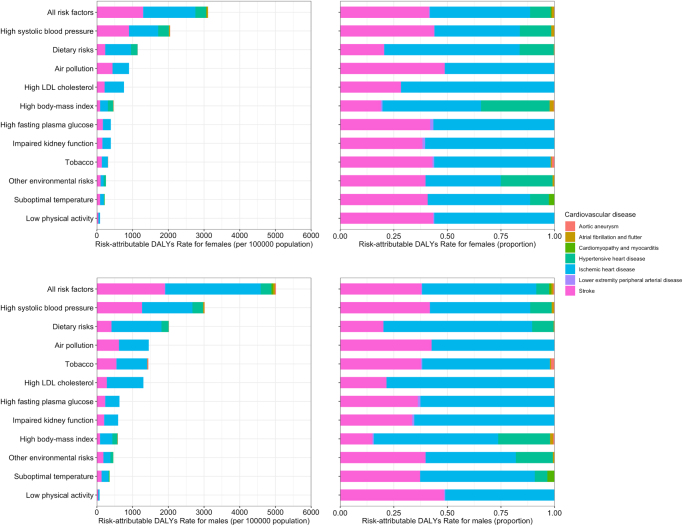
Risk-attributable DALYs rate for females and males in 2021.

As shown in Figure 5, high systolic blood pressure emerged as the primary contributor to CVD-related DALYs (301.8 million and 205.1 million, respectively) in both males and females, followed by dietary risks (202.0 million and 114.3 million, respectively). Tobacco use represented a significant risk factor for males (144.0 million), markedly contributing to the burden of ischemic heart disease and stroke (85.8 million and 54.3 million, respectively), whereas its impact was comparatively lower in females (31.5 million). Air pollution and elevated LDL cholesterol were also substantial contributors across both genders, exhibiting similar effects. Although both genders demonstrated a comparable hierarchy in the ranking of risk factors, males consistently presented with higher risk-attributable DALYs across nearly all risk categories. Elevated DALYs in males were particularly associated with tobacco use (144.0 million DALYs for males vs. 31.5 million for females), elevated LDL cholesterol (130.1 million DALYs for males vs. 75.9 million for females), and impaired kidney function (59.8 million DALYs for males vs. 39.3 million for females), indicating a greater burden of ischemic heart disease and related conditions. In contrast, females exhibited a higher proportional burden of stroke and hypertensive heart disease, predominantly linked to high systolic blood pressure and dietary factors.

## Discussion

In the present study, we not only investigated the global trend in the prevalence of CVDs, but also studied whether there is a statistically significant difference between genders during 1990–2021. Briefly, using data from GBD 2021, we demonstrated a substantially decreased overall CVD burden in men and women. However, our findings highlight significant sex differences in the burden of CVDs globally, with males experiencing a substantially higher burden across multiple metrics, including incidence, prevalence, mortality, and DALYs. The overall burden of CVDs, particularly for ischemic heart disease, intracerebral hemorrhage, and cardiomyopathy/myocarditis, was notably greater in males, driven by both biological factors and differential exposure to risk factors. Furthermore, women presented a higher prevalence of subarachnoid hemorrhage and pulmonary arterial hypertension, although without additional contribution to a higher mortality or DALY rates. These results suggest the need for differentiated cardiovascular risk prevention and management campaigns.

Our knowledge about sex differences in CVDs has been substantially advanced over the past decades; however, much of this knowledge awaits clinical adoption. Many previous studies have explored this issue^[4,5,11,12]^. Millett ERC *et al* reported that although the incidence of MI was higher in men, several risk factors were more strongly associated with MI in women compared with men[5]. Another study showed that females have higher prevalence rates of CVDs worldwide, such as stroke, hypertensive heart disease, rheumatic heart disease, non-rheumatic valvular heart disease, and endocarditis[11]. However, these studies are usually confined to a single CVD, or to a particular country or region. To the best of our knowledge, this research using the data from GBD 2021 is the most comprehensive study attempting to dissect the current knowledge on sex differences in multiple CVDs globally, as well as to identify knowledge gaps hindering the development of sex and gender informed CVD prevention and management, which could be informative for future research in this field.

In line with our findings, males consistently showed higher age-standardized incidence and prevalence rates for most cardiovascular conditions in previous studies^[4,13]^. Ischemic heart disease presented the largest disparity, with males showing significantly greater incidence and prevalence compared to females[5]. This difference underscores the urgency for enhanced preventive measures targeting ischemic heart disease in males, particularly since this condition accounted for the highest number of excess deaths and DALYs in males. Additionally, the high prevalence of ischemic stroke and intracerebral hemorrhage in males highlights the need for targeted intervention strategies, as these conditions collectively contribute to a significant proportion of CVD-related morbidity and mortality. Interestingly, a few conditions demonstrated higher burdens in females, indicating potential sex-specific pathophysiology or risk exposure. Lower extremity peripheral arterial disease was more prevalent in females, as was the burden of subarachnoid hemorrhage and pulmonary arterial hypertension^[14–19]^. These findings suggest a unique risk profile for females that warrants focused prevention and early diagnosis efforts, especially given the disproportionate impact of conditions such as subarachnoid hemorrhage, which are often associated with significant long-term disability.

Temporal trends between 1990 and 2021 reveal that, despite declines in age-standardized DALY rates for both sexes, males consistently experienced a higher burden of overall CVDs. Notably, the difference in DALY rates for conditions such as rheumatic heart disease and pulmonary arterial hypertension decreased over time, indicating potential success in interventions aimed at reducing disparities. However, the continued high burden in males, particularly for ischemic heart disease and ischemic stroke, suggests that the underlying drivers of these conditions-including risk factor exposure and healthcare access-remain inadequately addressed.

The reasons for sex differences in CVDs are not fully understood, but the protective effects of oestrogen on cardiovascular health in females are widely recognized[20]. Experimental studies have also shown that oestrogen plays a protective role in cardiovascular health by promoting angiogenesis and vasodilation, reducing oxidative stress and cardiac fibrosis, and elevating high-density lipoprotein cholesterol concentration[21]. However, clinical trials on exogenous oestrogen for postmenopausal women have shown no benefit for CVD risk and even increased risks for heart disease and venous thromboembolism[22]. A secondary analysis of the Women’s Health Initiative suggested oestrogen therapy could reduce coronary heart disease risk in women closer to menopause[23]. Therefore, the effects of sex hormones on CVD risks in females ought to be taken seriously and further explored in the present study.

Regional differences across socio-demographic index (SDI) levels highlight the link between socioeconomic factors and CVD burden. In this study, high SDI countries showed significant reductions in prevalence, incidence, and mortality due to better healthcare, preventive measures, and treatment access. In contrast, middle and low-middle SDI regions showed rising trends, driven by risk factors like hypertension, air pollution, low education, and limited healthcare access. Sex-related differences were minimal, suggesting that disparities in healthcare access and risk factor management similarly affect both sexes across these regions. However, a previous study demonstrated that the rates of major CVDs and death were substantially higher in low-income countries than in high-income countries, despite the fact that low-income countries had the lowest risk-factor burden[24]. This discrepancy is likely that the high burden of risk factors in high-income countries may have been mitigated by better control of risk factors and more frequent use of proven pharmacologic therapies and revascularization. These differentiated findings reinforce the importance of building intensive health care systems in middle and low-middle SDI regions, particularly in areas with constrained surgical infrastructure and high disease burden. To reduce the cardiovascular burden equitably, concrete public health strategies must be implemented, including the integration of gender-sensitive screening programs, primary prevention and sex-specific health promotion efforts.

In our current study, the analysis of risk factors associated with CVD-related DALYs demonstrated that most CVD risk factors were similar in women and men, and high systolic blood pressure, dietary risks, and air pollution were primary contributors for both sexes. However, the influence of risk factors such as tobacco use, elevated LDL cholesterol, and impaired kidney function was disproportionately higher in males, which aligns with the greater incidence of ischemic heart disease and stroke observed in this group. These findings support the need for gender-specific public health interventions aimed at reducing these modifiable risk factors, particularly among males. On the other hand, females showed a higher proportional burden of stroke and hypertensive heart disease, emphasizing the importance of targeted management of hypertension and dietary modifications in reducing CVD burden in women. These observations underscore the need to refine clinical practice guidelines by incorporating sex-specific approaches to cardiovascular screening—such as earlier and more frequent blood pressure and lipid evaluations in women, and intensified tobacco cessation support and cholesterol control strategies in men. Such tailored approaches could enhance preventive efficacy, improve patient adherence, and ultimately contribute to reduced sex disparities in cardiovascular outcomes. More notably, consistent with previous report, the similar associations of other risk factors with CVDs in women and men emphasized the importance of a similar strategy for the prevention of CVSs in both sexes[9].

CVD remains one of the leading contributors to perioperative morbidity and mortality, particularly in aging surgical populations. Understanding sex-specific differences in CVD burden and associated risk factors is directly relevant to preoperative risk stratification, perioperative management, and long-term outcomes after surgery. Moreover, as surgery is increasingly used to manage cardiovascular conditions (e.g., coronary revascularization, valve replacement, aortic repair), insights into upstream population-level disease burden can inform surgical service planning and policymaking. The findings have implications for global surgical system planning and resource allocation, particularly in middle and low-middle SDI regions where surgical capacity is limited, and where CVD burden may strain already overburdened perioperative services.

Several limitations should be considered in our study. First, better data related to the incidence of cardiovascular events, as well as the severity and associated disability of CVDs, are needed. Comorbidity of CVDs and the joint effects of multiple risk factors remain a topic that needs further investigation. Second, although we identified high systolic blood pressure and dietary risks as the dominant causal mediators for CVD-related DALYs in both females and males, several other risk factors, such as economic and lifestyle factors that play a key role in the development of CVDs. Thus, further studies focusing on gene-environment interactions may provide valuable insights into personalized prevention of cardiovascular risk. Third, although GBD 2021 adjusts the weight and variance accordingly, the use of disparate sub-national population-based studies may result in compositional biases in national estimates. Finally, it should be noted that while this study focuses on binary sex classifications as reported in the GBD database, it does not capture gender identity data, including populations such as transgender, non-binary, and other sexual and gender minorities. Although beyond the scope of the current analysis, the cardiovascular risk profiles and healthcare disparities affecting these populations represent a critical and underexplored dimension of CVD research. Future studies should prioritize the inclusion of gender-diverse populations to better inform equitable public health strategies and tailored clinical interventions.

## Conclusion

In summary, our study showed that CVD prevalence varies by sex, highlighting the need for gender-specific prevention and management strategies, particularly in aging populations. Public health efforts should address key risk factors while accounting for sex differences and prioritize strengthening healthcare in middle and low-middle SDI regions to reduce disparities. Integrating population-level CVD insights into surgical decision-making and healthcare policy will be essential to improving cardiovascular outcomes globally.

## Data Availability

The data supporting the findings of this study are openly available in the GBD 2021 results (https://vizhub.healthdata.org/gbd-results/). Reasonable access to all codes that produce secondary results in this study can be obtained by contacting the corresponding author (J.Q.F., jqfan@zju.edu.cn).
